# 8-oxoguanine and 8-oxodeoxyguanosine Biomarkers of Oxidative DNA Damage: A Review on HPLC–ECD Determination

**DOI:** 10.3390/molecules27051620

**Published:** 2022-03-01

**Authors:** Ana-Maria Chiorcea-Paquim

**Affiliations:** 1 University of Coimbra, Centre for Mechanical Engineering, Materials and Processes (CEMMPRE), Department of Chemistry, 3004-535 Coimbra, Portugal; anachior@ipn.pt; 2 Instituto Pedro Nunes (IPN), 3030-199 Coimbra, Portugal

**Keywords:** 8-oxoguanine (8-oxoG), 8-oxo-2′-deoxyguanosine (8-oxodG), oxidative DNA damage, oxidative DNA damage biomarker, HPLC–ECD

## Abstract

Reactive oxygen species (ROS) are continuously produced in living cells due to metabolic and biochemical reactions and due to exposure to physical, chemical and biological agents. Excessive ROS cause oxidative stress and lead to oxidative DNA damage. Within ROS-mediated DNA lesions, 8-oxoguanine (8-oxoG) and its nucleotide 8-oxo-2′-deoxyguanosine (8-oxodG)—the guanine and deoxyguanosine oxidation products, respectively, are regarded as the most significant biomarkers for oxidative DNA damage. The quantification of 8-oxoG and 8-oxodG in urine, blood, tissue and saliva is essential, being employed to determine the overall effects of oxidative stress and to assess the risk, diagnose, and evaluate the treatment of autoimmune, inflammatory, neurodegenerative and cardiovascular diseases, diabetes, cancer and other age-related diseases. High-performance liquid chromatography with electrochemical detection (HPLC–ECD) is largely employed for 8-oxoG and 8-oxodG determination in biological samples due to its high selectivity and sensitivity, down to the femtomolar range. This review seeks to provide an exhaustive analysis of the most recent reports on the HPLC–ECD determination of 8-oxoG and 8-oxodG in cellular DNA and body fluids, which is relevant for health research.

## 1. Introduction

**Scheme 1:**
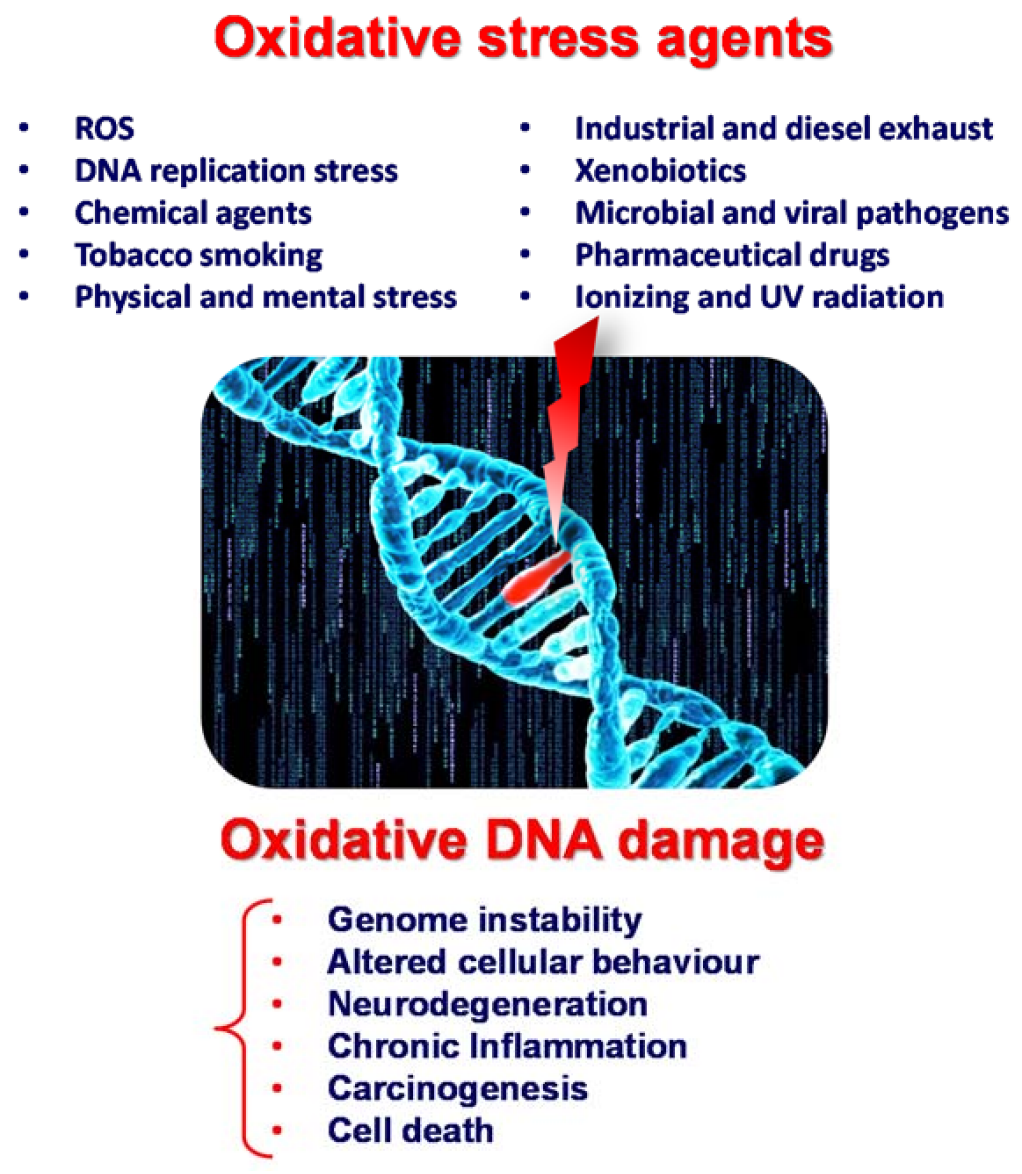
Schematic representation of oxidative stress agents and oxidative DNA damage.

Reactive oxygen species (ROS) are produced endogenously due to cellular metabolism or exogenously due to exposure to physical, chemical, or biological agents such as γ, X-ray and UV radiation, tobacco smoking, pollutants, toxins, xenobiotics, and bacterial and viral pathogens; see Scheme 1. Although ROS exhibit a beneficial role at the cellular level, being involved in inter- and intracellular signalling, excessive ROS cause oxidative stress and lead to more than 100 different types of oxidative DNA lesions in mammals, including DNA base oxidation, double- and single-strand breaks, intra- and interstrand crosslinks, and abasic sites.

Leaving oxidative DNA damage unrepaired results in changes or mutations within the cell genomic material, leading to genomic instability, changes in gene expression, altered cellular behaviour, neurodegeneration, chronic inflammation, carcinogens and (ultimately) cell death; see Scheme 1. Hence, many diseases are correlated with oxidative DNA damage, e.g., neurodegenerative, cardiovascular, and autoimmune inflammatory diseases; diabetes; cancer; and other age-related diseases.

**Scheme 2:**
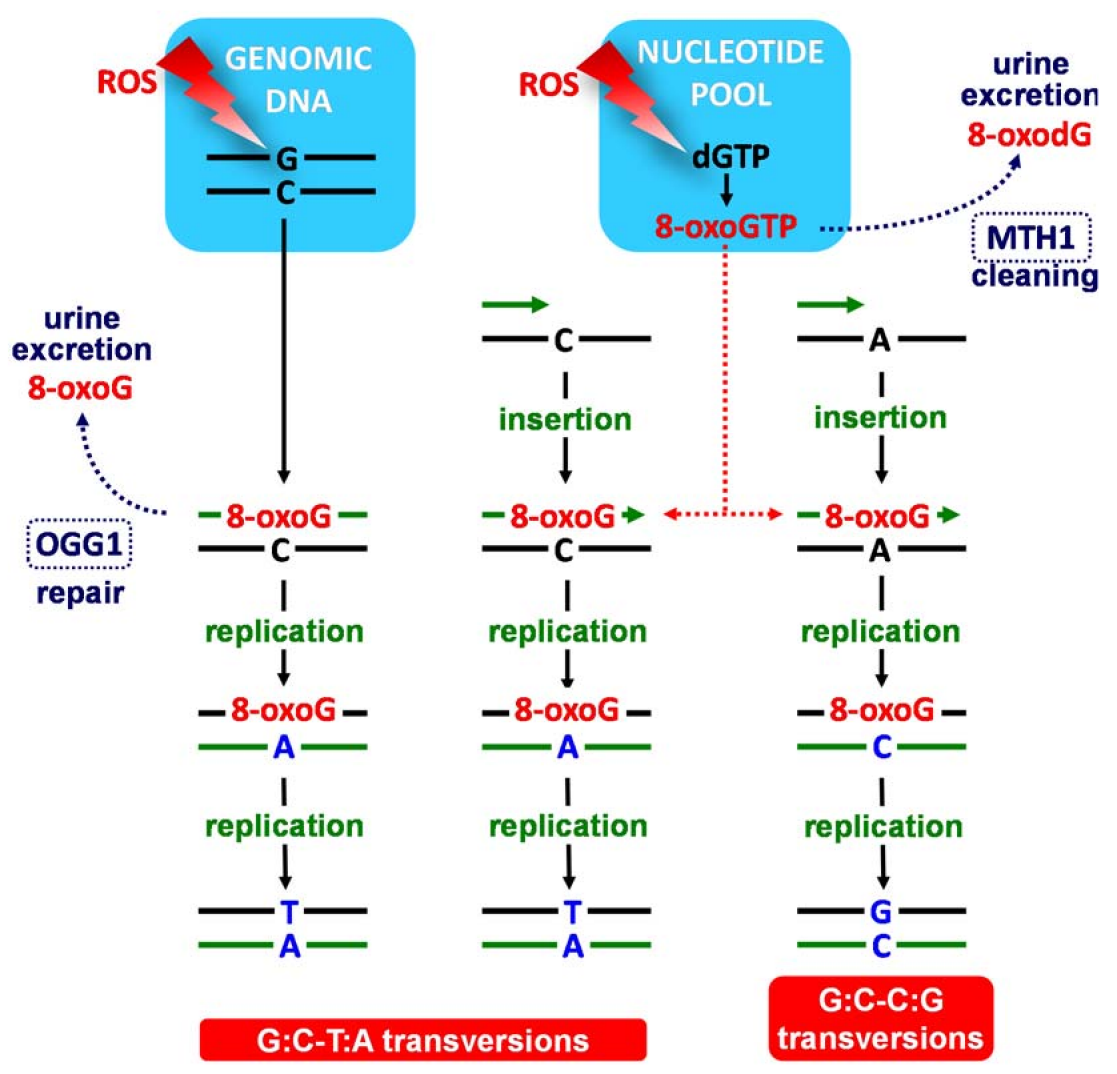
Schematic representation of mutagenesis caused by 8-oxoguanine (8-oxoG), the major base lesion in DNA caused by ROS that leads to G:C–T:A and G:C–C:G or transversions. Black and green lines represent the template DNA strand and the nascent DNA strand, respectively, during replication.

The oxidation of the DNA bases by ROS can occur either directly in the genomic DNA strands or indirectly in the nucleotide pool, from where the modified bases are introduced into the genomic DNA during replication or repair; see Scheme 2 [1,2,3].

**Scheme 3:**
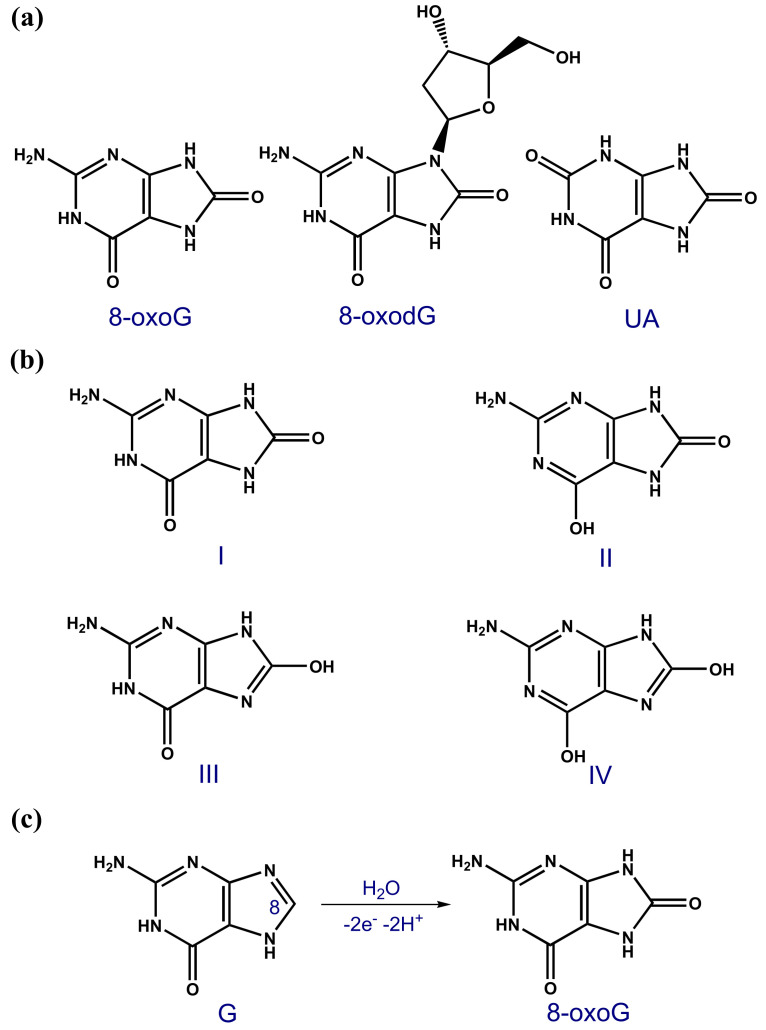
(**a**) Chemical structures of 8-oxoguanine (8-oxoG), 8-oxo-2′-deoxyguanosine (8-oxodG), and uric acid (UA); (**b**) 8-oxoG tautomers; and (**c**) the oxidation of guanine (G) to 8-oxoG.

The guanine (G) base is easier oxidised than adenine (A), cytosine (C) and thymine (T) [4], so its oxidation products are the most common forms of oxidative DNA damage [5,6,7]. 8-oxoguanine (8-oxoG or 8-oxoGua), also named 7,8-dihydro-8-oxoguanine, 8-dihydroguanine, or 8-hydroxyguanine (8-OHG); see Scheme 3a [8], the G oxidation product at the C8 position; see Scheme 3c, and its nucleotide 8-oxo-2′-deoxyguanosine (8-oxodG), also named 8-oxo-7,8-dihydro-2′-deoxyguanosine or 8-hydroxy-2′-deoxyguanosine (8-OHdG); see Scheme 3a, are notable biomarkers used for the quantification of oxidation stress and oxidative DNA damage in animals and humans.

**Scheme 4:**
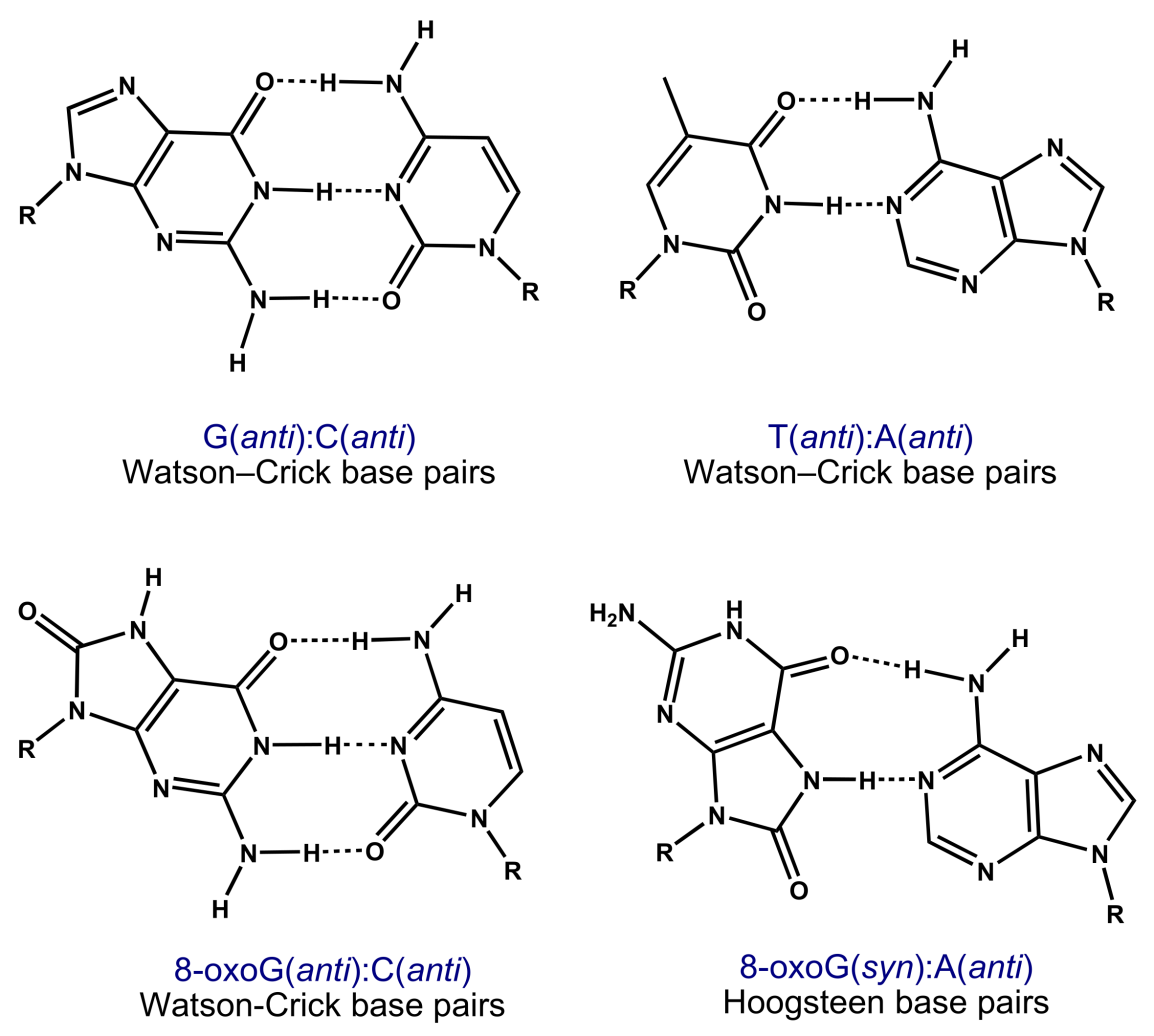
Structures of normal G(*anti*):C(*anti*) and T(*anti*):A(*anti*) Watson–Crick, and 8-oxoG(*anti*):C(*anti*) Watson–Crick and 8-oxoG(*syn*):A(*anti*) Hoogsteen base pairs.

The 6,8-diketo I form of 8-oxoG, which presents an H atom at the N7 position and an oxo group at the C8 position, was predicted to be the most stable tautomer; see Scheme 3b [9,10,11,12]. The high mutagenic capacity of 8-oxoG is related to its ability to form (i) Watson–Crick base pairs with C (Scheme 4) leading to G:C–C:G transversions (Scheme 2) and (ii) Hoogsteen base pairs with A (Scheme 4) leading to G:C–T:A transversions in the course of the DNA replication (Scheme 2) [3,5].

The cellular defence system against 8-oxoG within a DNA strand involves the base excision repair (BER) that corrects small base lesions [11]; it begins with the oxoguanine glycosylase 1 (OGG1) that identifies and eliminates 8-oxoG, which is then excreted in urine; see Scheme 2 [2]. The nucleotide pool is sanitized by the nucleoside diphosphate linked moiety X type motif 1 (MTH1 or NUDT1) that hydrolyses 8-oxodGTP to 8-oxodGMP, leading to 8-oxodG and/or 8-oxodG excretion in urine; see Scheme 2 [2]. Thus, the urinary excretion of 8-oxoG and 8-oxodG from damaged DNA and the nucleotide pool reflects the equilibrium between in vivo oxidative DNA damage and the efficiency of the cellular repair processes. Apart from urinary excreted 8-oxodG/8-oxodG, significantly increased levels of 8-oxodG/8-oxodG in cancerous tissue DNA than in corresponding normal tissues has been observed, especially for individuals with impaired OGG1 repair function that exhibit increased disease aggressiveness.

Therefore, the possibility of quantifying 8-oxoG and/or 8-oxodG in urine, blood, tissue and saliva has received a growing interest; both 8-oxoG and/or 8-oxodG are regarded as the most significant biomarkers for oxidative DNA damage, being used to determine the body oxidative stress and to evaluate the risk, diagnose, and predict the treatment impact of various diseases.

The analytical techniques generally employed for the quantification of 8-oxoG and 8-oxodG levels in biological matrixes are high-performance liquid chromatography (HPLC) with spectrophotometric detection [11] and electrochemical detection (ECD), HPLC–ECD [13,14], HPLC tandem mass spectrometry (HPLC–MS) [15], gas chromatography–mass spectrometry (GC–MS) [14,16], enzyme-linked immunosorbent assay (ELISA) [11,17], and (more recently) electrochemical sensors [18]. Among these methods, HPLC–ECD is highly preferred due to its selectivity and high sensitivity, down to the femtomolar range.

This review seeks to provide an exhaustive analysis of the most recent advances on the determination of 8-oxoG and 8-oxodG biomarkers in cellular DNA and body fluids (urine, blood serum and saliva) using HPLC–ECD, which is relevant for health research.

## 2. Determination of 8-oxoG and 8-oxodG by HPLC–ECD

Early studies mostly concerned the quantification of 8-oxodG. The first report in 1986 described the use of a gradient reversed-phase HPLC coupled with ECD to quantify 8-oxodG in DNA hydrolysates at the sub-picomole levels [13]. The ECD analytical determination was revealed to be 1000 times more sensitive than the UV detection method [19], which made the quantification of 8-oxodG possible in HeLa cells [20], mouse liver [20], rat kidney [21,22], rat liver [23,24], rat heart [25], and rat urine [21], as well as in human leukocytes [26], blood mononuclear cells [27], embryonic cells [11], lung [28], brain [29], breast [27,30], colorectal tumour tissue [31], and urine [32,33,34,35].

Before 2000, hundreds of studies reported DNA damage in animal organs after the administration of various carcinogenic chemical substances and tumour promoters. The 8-oxodG content in human organs, leucocyte DNA, and urine have been analysed and correlated with various diseases, aging, exposure to ionizing radiation, and lifestyle parameters (e.g., smoking, exercise, and ingestion of antioxidants) [36].

To facilitate the HPLC–ECD analysis of 8-oxoG and 8-oxodG in urine, plasma, cerebrospinal fluid, saliva, leukocytes and tissue samples, different purification methods have been elaborated, e.g., solid-phase extraction (SPE) [33,34,37], and purification in different types of columns: reversed-phase [19,38,39], carbon [40], cation-exchange [38,41], and anion-exchange [39,42,43] columns, coupled-columns (such as anion-exchange coupled with reversed-phase [44]), multifunction columns (such as with gel filtration, reversed-phase and cation-exchange [45]), and columns enriched with polyclonal [46] or monoclonal antibodies [47,48].

Concerning electrochemical conditions, the ECD detection of 8-oxodG initially employed fixed potentials between +0.10 and +0.40 V [48,49,50,51]. Later, the oxidation of other DNA components with similar retention times at ~0.38 V was observed, which led to 8-oxodG overestimation. Therefore, an optimum applied potential of ~ +0.25 V was found to reduce overlapping peaks and resolve the 8-oxodG overestimation problem [52]. The HPLC–ECD detection of 8-oxoG has generally been less employed than 8-oxodG, although 8-oxoG presents a lower oxidation potential than 8-oxodG [53,54,55,56].

### 2.1. Determination of 8-oxoG and 8-oxodG in DNA

8-oxodG determination in DNA from leukocytes and tissues samples requires a preliminary step of hydrolysis (usually enzymatic) to break down the DNA prior to HPLC separation and electrochemical (amperometric, coulometric, or voltammetric) detection. The results are usually given in terms of the molar ratio of detected 8-oxodG per 10^5^ undamaged deoxyguanosine (8-oxodG/10^5^ dG) [57]. In this context, the HPLC–ECD technique has shown two methodological problems: (1) the occurrence of the artefact oxidation of unmodified G bases during the DNA isolation and hydrolysis steps, especially in the case of enzymatic digestion, leading to the overestimation of 8-oxodG levels [58], and (2) the incomplete hydrolysis that leads to the underestimation of the oxidative damage. Both problems have resulted in a lack of consistency in the results among different laboratories, especially concerning the overestimation of the 8-oxodG background levels in human cellular DNA [59,60].

Different factors influencing the DNA isolation and sample processing for HPLC–ECD have been evaluated, such as (1) the utilization of phenol, (2) the utilisation of chaotropic techniques, (3) the examination of very small DNA concentrations (less than 20 µg), (4) the DNA hydrolysis time, (5) the existence of redox-active metals, (6) the parameters used in the DNA extraction, (7) the chromatographic interference, (8) the adduct recovery during immunoaffinity purification, and (9) the formation of oxidized G derivatives by xanthine oxidase [49]. The use of phenol has been particularly discussed, as it may act as pro-oxidant during DNA extraction [49,61]. Other artefacts could be associated with the prolonged digestion of proteins during DNA extraction and prolonged DNA hydrolysis [49].

To solve the HPLC–ECD issues, especially the artefact oxidation occurring during isolation and purification, The European Standards Committee on Oxidative DNA Damage (ESCODD), with 27 analytical laboratories as members, was established [62]. The 8-oxodG levels in 8-oxodG standard samples, calf thymus DNA, oligonucleotides, HeLa cells and blood lymphocytes have been tested [59,60,62,63,64,65]. Based on controlled methodologies, coulometric and amperometric HPLC–ECD has been used to determine the 8-oxodG/10^5^ dG ratio, and the results have been compared with the HPLC–MS/MS and GC–MS results, and mean values among all techniques were established [65,66,67].

Different methods have been proposed and many protocols have been revised to reduce the above-mentioned problems, including (i) the development of non-phenol DNA extraction methods [11,49,50,51,68] (e.g., chaotropic NaI [49] or DNAzol protocols [68]), (ii) the limitation of the incubation time [69], (iii) the use of cold workup procedures [50,69], and (iv) the use of antioxidants and metal chelators during sample preparation [49,52,70,71]. The use of phenol has been particularly controversial, although different studies showed that phenol extraction may not be responsible for 8-oxodG overestimation, which is instead caused by the sample impurities combined with excessive air exposure [49,69]. The use of desferrioxamine (brand name Desferal^®^), which chelates traces of redox-active iron, has been found to reduce the problems related with the duration of the hydrolysis process and protein digestion during DNA extraction [49,71], and the use of NaI or guanidine thiocyanate has been found to improve the problems related with DNA precipitation [50,71]. The use of careful HPLC hygiene during analysis helped reducing the artefacts resulting from the analysis of very small quantities of DNA [49].

8-oxodG levels in human leukocytes were used to assess oxidative DNA damage and found to be correlated with smoking, physical exercise, and alcohol consumption [72]. The mean molar 8-oxodG/10^5^ dG ratio was significantly higher in smokers (33.1 ± 10.6) compared to non-smokers (15.3 ± 1.8) and former smokers (17.8 ± 1.5), the highest values being observed for individuals smoking over 10 cigarettes per day (41.8 ± 17.1). The frequency of physical exercise or alcohol drinking did not significantly modify 8-oxodG levels in leukocytes [72]. In a different report, lower mean 8-oxodG/10^5^ dG levels in leukocytes—2.86 ± 1.39 in smokers and 2.72 ± 1.36 in non-smokers—were obtained [73].

Vitamin C’s effects against intracellular ROS production were investigated using HPLC–ECD with a modified pronase/ethanol extraction method [74], and it was shown that the mean 8-oxodG/10^6^ dG ratio in the cellular DNA of peripheral blood lymphocytes decreased from 22.9 to 18.8 following 8 weeks of vitamin C supplementation. The decrease was also observed following vitamin C supplementation in four subgroups: (1) from 17.2 to 14.6 in subjects with less than 500 µg L^−1^ of ferritin, (2) from 29.1 to 23.3 in subjects with more than 500 µg L^−1^ of ferritin, (3) from 21.6 to 17.2 in subjects with a transferrin saturation (TSAT) of less than 50% and (4) from 23.8 to 17.8 in subjects with a TSAT of above 50%, but no significant modifications were observed in subjects treated with a placebo [74]. In a different report, using a purification method of genomic DNA from human whole blood via isopropanol-fractionation with concentrated NaI and sodium dodecyl sulphate, it was shown that 80, 200, and 400 mg of vitamin C supplementation had little effect on healthy individuals with diets abounding in vitamin C, with the mean 8-oxodG/10^6^ dG levels remaining unaltered [75].

The 8-oxodG levels in human leukocytes have been used to determine of the role played by oxidative stress in the pathogenesis of Leber’s hereditary optic neuropathy (LHON) [76]. The mean 8-oxodG/10^5^ dG ratio was found to be 1.34 ± 0.99 in patients with LHON with an 11778 mitochondrial DNA mutation, 1.00 ± 0.91 in their asymptomatic maternal relatives, and 0.31 ± 0.20 in normal control subjects, which suggested that the LHON phenotype might be the result of an increase in mitochondrial ROS caused by LHON mutations.

The increase in the oxidative DNA damage in living kidney donors, three years post-donation, was assessed, and a direct association between the pre-donation serum urea and creatinine and post-donation 8-oxodG/dG ratio was observed [77]. The study demonstrated a positive correlation between pre-donation serum urea and creatinine and post-donation oxidative DNA damage (8-oxodG/dG ratio) in kidney donors.

The 8-oxodG/10^5^ dG ratio in different types of cancer tissues has been determined [78], e.g., breast cancer (2.07 ± 0 95 in cancer tissue vs. 1.34 ± 0 46 in control [79] and 10.7 ± 15.5 in cancer tissue vs. 6.3 ± 6.8 in control surrounding tissue [80]) and colorectal cancer (2.53 ± 0.15 in cancer tissue vs. 1.62 ± 0.13 in control [81], 49 in cancer tissue vs. 21 in control [82], 1.34 ± 0.11 in cancer tissue vs. 0.64 ± 0.05 in control [31], and 2.4 ± 1.1 in cancer tissue vs. 1.6 ± 0.6 in control [83]).

Different studies have reported changes in the mean 8-oxodG/10^5^ dG ratios in cellular DNA induced by various environmental factors: (i) ambient particulate air pollution assessed as outdoor concentrations of particulate matter ≤2.5 μm in diameter (PM2.5) (0.55 in autumn, 0.27 in winter, 0.62 in spring, and 0.58 in summer, as determined in lymphocytes) [84], (ii) asbestos (2.61 ± 0.91 in individuals exposed in 1994–1995, 2.96 ± 1.10 in individuals exposed in 1995–1996, 2.55 ± 0.56 in individuals exposed in 1996–1997 vs. 1.52 ± 0.39 in unexposed controls, as determined in leukocytes) [85,86], (iii) silica (2.51 ± 1.36 in silicotics and 3.20 ± 2.25 in exposed workers, as determined in leukocytes) [87], (iv) polycyclic aromatic hydrocarbon (PAH) (1.24 ± 0.70 at the start and 1.57 ± 1.65 at the end of the week in roofers exposed to asphalt fume, 1.03 ± 0.71 at the start and 0.56 ± 0.24 at the end of the week in roofers exposed to coal-tar, 1.96 ± 0.83 at the start and 1.96 ± 0.83 at the end of the week in unexposed controls [88], and 4.31 at bottom, 3.08 at middle, and 3.07 at top PAH exposure in coke-oven workers at an iron-steel factory [89]), (v) KBrO [90], (vi) chloroform [90], (vii) bromodichloromethane [90], (viii) pulmonary carcinogen 4-(methylnitrosamino)-1-(3-pyridyl)-1-butanone (NNK) found in tobacco [91], (ix) mycotoxin aflatoxin B1 (AFB1) produced by Aspergillus fungi [92], and (x) γ-ray irradiation [93].

### 2.2. Determination of 8-oxoG and 8-oxodG in Body Fluids

The HPLC–ECD method of 8-oxoG and 8-oxodG determination in urine presents several advantages [94]: (i) the collection of urine samples is less invasive, (ii) the artifacts induced by the DNA isolation and sample processing steps are minimized, and (iii) 8-oxoG and 8-oxodG are very stable in urine, mimicking the equilibrium between oxidative DNA damage and cellular repair processes.

Later on, an European Standards Committee for Urinary (DNA) Lesion Analysis (ESCULA) was established to validate the assays and the value ranges of 8-oxodG in urine [95,96,97]. For urinary 8-oxodG biomarker detection, a correction of urine concentration is made, and the results are generally given as a ratio of 8-oxodG/creatinine concentration [95,98,99].

**Figure 1:**
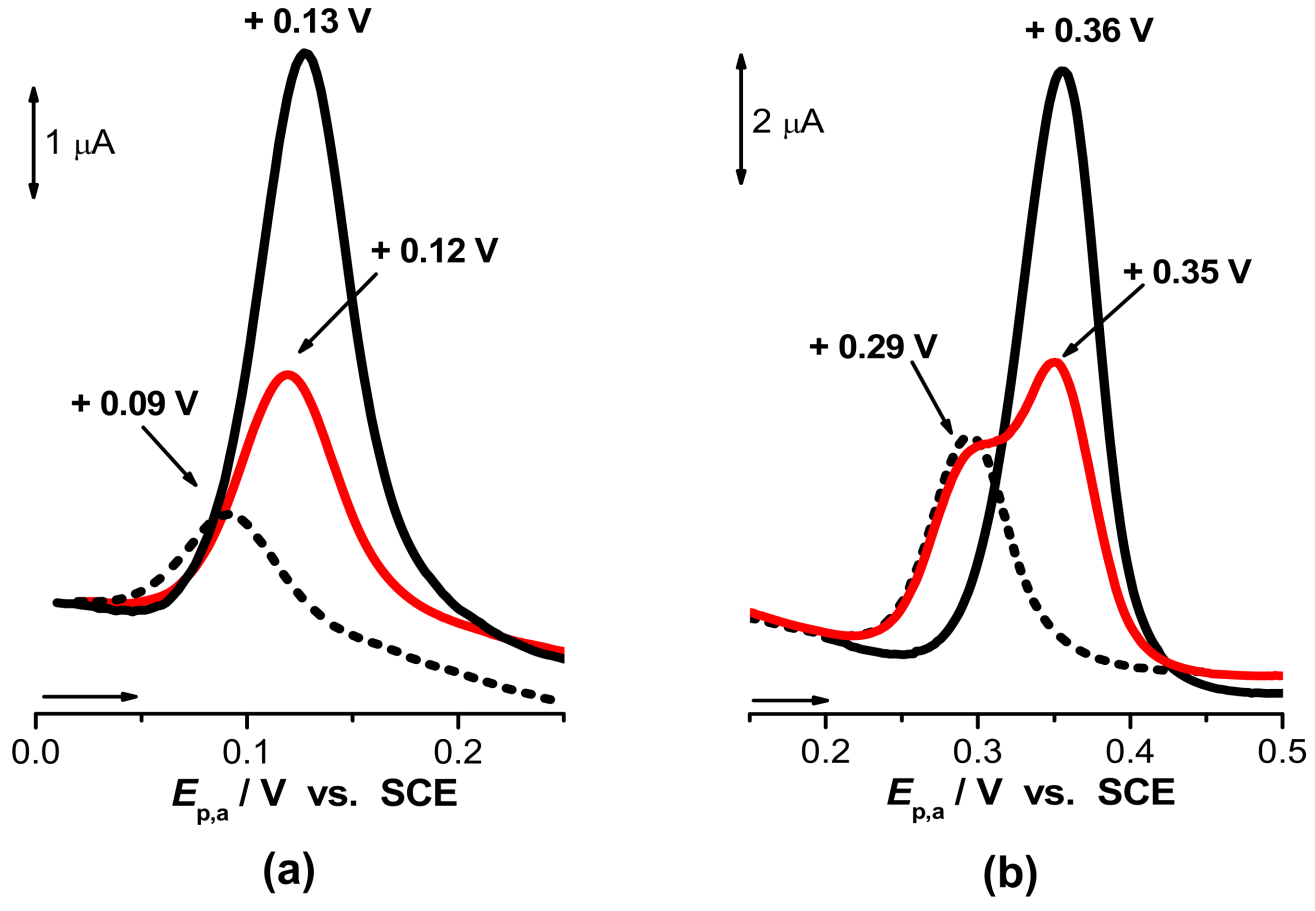
Differential pulse voltammograms at a glassy carbon electrode in a 5 µM solution of (black line) 8-oxoG, (black dotted line) UA, and (red line) a 5 µM equimolar mixture of 8-oxoG and UA in (**a**) pH = 9.4 and (**b**) pH = 6.2. Reproduced from [102] with permission. Copyright 2004 Elsevier.

The separation of 8-oxoG and 8-oxodG from constituents of the urine matrix represents the most important challenge in the development of precise analytical determination methods, and different methodologies have been described [54,100,101]. In human urine, the uric acid (UA) concentration is 10^4^-fold higher than the basal levels of 8-oxoG and 8-oxodG. 8-oxoG and UA present comparable chemical structures (Scheme 3), and the difference between their oxidation potentials is less than 0.1 V across the entire pH range; see Figure 1 [102]. In these conditions, 8-oxoG and 8-oxodG detection is especially difficult due to the proximity of their retention times.

Differential pulse voltammograms at a glassy carbon electrode showed that the best separation between the 8-oxoG and UA oxidation peaks was achieved for pH = 6.0; see Figure 1b [102].

**Figure 2:**
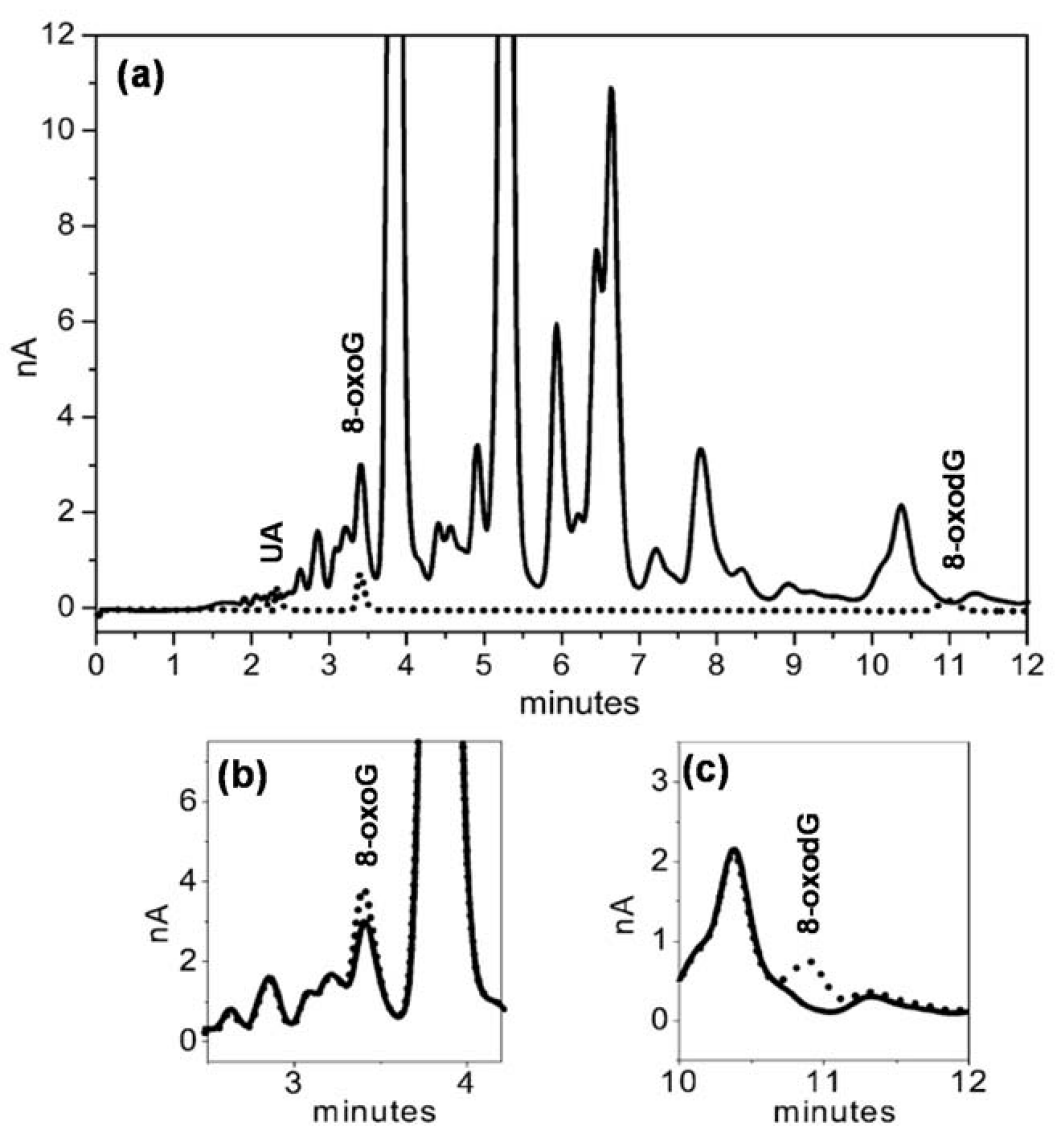
(**a**) HPLC chromatograms obtained from (black dotted line) a 10 µL injection of a 100 nM standard mixture of UA, 8-oxoG, and 8-oxodG; (black line) a 10 µL injection of a urine sample from a child with metabolic disorders after being processed by solid phase extraction (SPE). (**b**,**c**) Same urine sample (black line) before and (black dotted line) after being spiked with 250 nM standard solutions of (**b**) 8-oxoG and (**c**) 8-oxodG. Reproduced from [54] with permission. Copyright 2004 Elsevier.

Based on these results, an HPLC–ECD method for the simultaneous detection of 8-oxoG and 8-oxodG with a good separation factor (α = 3.7) in excess UA conditions was developed [54]. This method consists of an HPLC isocratic elution with amperometric detection on a GCE electrode, enabling a limit of detection (LOD) for 8-oxoG and 8-oxodG lower than 1 nM in standard mixtures; see Figure 2. The detection of 25 nM concentrations of 8-oxoG and 8-oxodG in solutions containing 10^4^-fold higher concentration of UA was achieved after one-step SPE and enhanced by uricase digestion. The method was tested in urine samples from children with metabolic disorders (see Figure 2) and adults with cognitive deficits, showing an LOD for 8-oxoG of 80 nM [54].

**Figure 3:**
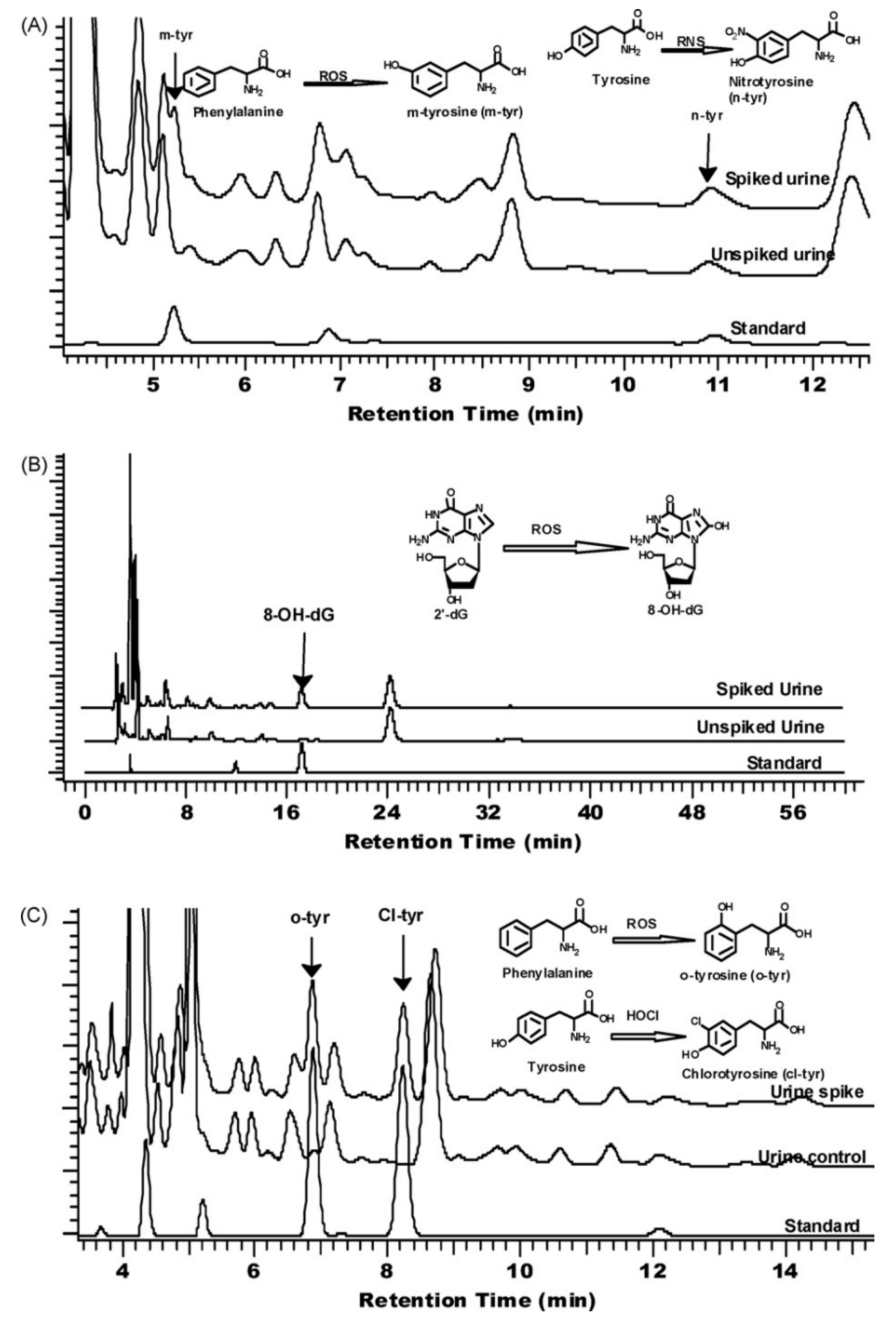
Identification of target analytes in spiked urine samples at different electrochemical channels: (**A**) m-tyrosine and nitrotyrosine (0.65 V); (**B**) 8-oxodG (0.25 V); (**C**) o-tyrosine and cl-tyrosine (0.50 V). Reproduced from [103] with permission. Copyright 2010 Elsevier.

Based on a cleaning step by anion-exchange chromatography and a purification step by reversed-phase HPLC–ECD, the simultaneous determination of urinary 8-oxodG and creatinine [42] or 7-methylguanine [43] has been carried out. Moreover, based on a solid phase extraction method using a reversed-phase polymeric sorbent column prior to HPLC–ECD analysis, the simultaneous analysis of urinary oxidative/nitrative stress biomarkers o-, m-, cl-, 3-nitro-tyrosine, and 8-oxodG in human urine was achieved; see Figure 3 [103].

Different studies have reported baseline urinary 8-oxodG concentrations in healthy adults [94], e.g., 3.16 ± 1.28 µg/g of creatinine in men [104], 2.81 ± 1.07 µg/g of creatinine in men [105], 3.04 ± 1.42 µg/g of creatinine in women [105], 6.3 ± 0.5 µmol/mol of creatinine in men [106], 1.62 ± 0.50 µmol/mol of creatinine in man and women [107], and 4.70 ± 7.1 µmol/mol of creatinine in women [108,109].

**Figure 4:**
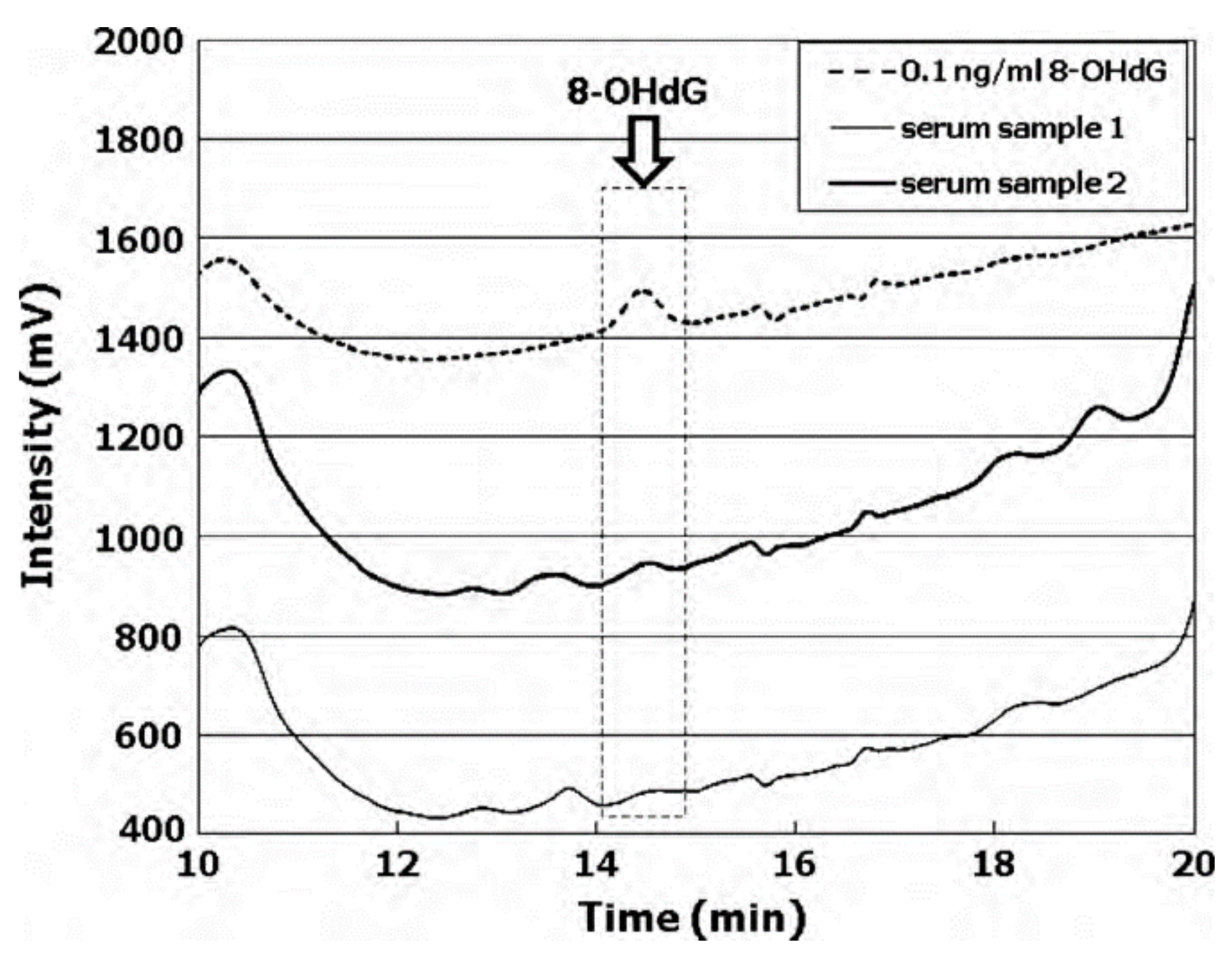
HPLC chromatograms of serum samples for healthy people. HPLC conditions were a mobile phase consisting of a 35 mmol L^−1^ phosphate buffer (pH 7.0) containing 5% acetonitrile and 30 mol L^−1^ EDTA, a column oven temperature of 27 °C, an injection volume of 100 L, a flow rate of 1.0 mL/min, and an HPLC Develosil C30 prepacked column (5 m particle size). Reproduced from [37] with permission. Copyright 2010 Elsevier.

Based on an SPE method consisting on 8-oxodG separation from urine [100] and blood serum [37] in C18 and strong cation-exchange (SCX) columns, the most significant interfering compounds have been eliminated. For 8-oxodG in serum, an LOD in the control group of less than 10 pg mL^−1^ was achieved, eliminating the necessity for sample evaporation. The 8-oxodG levels in the sera from healthy individuals showed a mean value of 25.5 ± 13.8 pg mL^−1^; see Figure 4 [37].

8-oxoG levels measured in urine and serum showed a substantial increase in type 2 diabetic db/db mice when compared to control db/m+ mice, but no change in the 8-oxodG levels in the liver and kidney DNA was observed, suggesting that urinary and serum 8-oxodG/8-oxoG are better biomarkers of oxidative stress [98].

Employing the use of monoclonal antibodies [47], the serum 8-oxoG levels in type 2 diabetic patients was assessed and showed significantly higher concentrations (5.03 ± 0.69 nM) compared to control individuals (0.96 ± 0.15 nM) [110]. Moreover, among diabetic patients, those with proliferative retinopathy had significantly higher 8-oxoG levels (8.27 ± 0.31 nM) than those with non-proliferative retinopathy (4.92 ± 0.34 nM) or without retinopathy (3.38 ± 0.22 nM), but no correlation between the 8-oxoG level and duration of diabetes was established.

The influence of lifestyle factors on the urinary levels of 8-oxodG was determined [45,111,112,113]: the 8-oxodG level decreased with moderate physical exercise of less than 5 h/week (3.45 µg/g of creatinine) and high body mass index (BMI) values (3.41 µg/g of creatinine), while its level increased with physical labour (4.32 µg/g of creatinine), smoking (3.94 µg/g of creatinine), and low meat intake (less than once per week; 4.56 µg/g of creatinine) [45].

The urinary 8-oxodG level was also found to be directly correlated with high blood sugar levels (3.40 µg/g of creatinine) and leanness in smokers (3.40 µg/g of creatinine for BMI > 25 kg/m^2^ vs. 4.35 µg/g of creatinine for BMI < 18 kg/m^2^) [111]. Moreover, urinary 8-oxodG increased with exposure to organic solvents and hydrochloric acid (3.75 µg/g of creatinine) and long working hours habits (5.05 µg/g of creatinine). The urinary 8-oxodG level was found to be negatively correlated with high LDL cholesterol (3.05 µg/g of creatinine) [111], total cholesterol [114], and anaemia (1.40 µg/g of creatinine) [111]. At the individual level, no differences were detected in the diurnal urinary 8-oxodG levels, but changes have been observed with changes in lifestyle factors, e.g., exercise, sleep duration, drinking habits, diet, and stress [115,116], while depressive symptoms did not present a significant correlation with 8-oxodG [116].

In subjects enrolled in a quit-smoking program, the 8-oxodG levels decreased from 5.21 µg/g of creatinine before to 4.75 µg/g of creatinine after 2 weeks and 5.09 µg/g of creatinine after 8 weeks after the cessation of smoking, following decreases in the smoking exposure markers of nicotine, cotinine and 4-(methylnitrosamino)-1-(3-pyridyl)-1-butanol (NNAL) [117].

Species with short potential life spans usually present higher metabolic rates correlated with higher oxidative DNA damage. The urinary 8-oxoG levels from mammals with different life spans (mice, rats, guinea pigs, cats, chimpanzees, and humans) showed a negative correlation with their potential life spans, while 8-oxodG showed only a slight tendency toward a negative correlation, which suggests that 8-oxoG may be a better overall biomarker for oxidative DNA damage than 8-oxodG [118,119].

The possibility of prediction of cancer risk was evaluated. Using an HPLC–ECD approach based on anion-exchange chromatography, precise fraction collection, and reversed-phase chromatography, it was established that 8-oxodG excretion in urine increased for on-going smokers (1.94 nmol/mmol of creatinine in subjects with lung cancer vs. 1.81 nmol/mmol of creatinine in controls), whilst a high 8-oxodG excretion among never-smokers was correlated with a higher risk of lung cancer development [39]. In a similar study, the urinary 8-oxodG/creatinine ratio was found to be significantly increased in smoking subjects (1.95 ± 0.40 µmol/mol) compared to non-smoking subjects (1.62 ± 0.50 µmol/mol) [107]. Moreover, high levels of the urinary 8-oxoG biomarker were correlated with higher lung cancer risk in men, never-smokers, and former smokers [120].

The urinary 8-oxodG levels of breast cancer patients at each stage of carcinogenesis were determined, and the researchers reported higher values of 16.39 ± 17.3 µmol/mol of creatinine in the breast cancer group compared to the 4.70 ± 7.1 µmol/mol of creatinine in the control group [108,109].

Many studies have reported comparative analyses of the 8-oxodG levels in both urine and cellular DNA. The HPLC–ECD detection of urinary 8-oxodG was used as a predictor for acute radiosensitivity in breast cancer patients [121], and both urinary 8-oxoG and leukocytes 8-oxodG biomarkers were used to predict the clinical success of radiotherapy treatment [122], due to the fact that high levels of urinary 8-oxoG and concomitant stable levels of 8-oxodG in leukocyte DNA were demonstrated to be associated with lower risks of mortality during the course of treatment.

The 8-oxodG levels in urine and in peripheral mononuclear cells (PMNC) from gastric cancer patients and healthy control subjects, before and after tumour resection, were analysed, and it was shown that the 8-oxodG/10^6^ dG ratio was significantly higher in the PMNC of gastric cancer patients (4.16 ± 0.73) compared to healthy control individuals (8.43 ± 1.3), which was also supported by the urinary 8-oxodG levels in gastric cancer patients (22.29 ± 4.79 nmol/mmol of creatinine) and controls (2.49 ± 1.07 nmol/mmol of creatinine) [99]. After gastrectomy, the 8-oxodG levels slowly decreased to values close to those of healthy subjects, suggesting that high 8-oxodG urinary levels could be associated with increased DNA repair activity in tumour tissues.

Patients with monoclonal B lymphocytosis (MBL) and chronic lymphocytic leukaemia (CLL) showed a significant increase in 8-oxodG levels in the lymphocytes (8-oxo-dG/10^6^ dG ratio of 39.5 in MBL and 48.4 in CCL vs. 6.1 in control) and urine (nmol 8-oxodG/mmol of creatinine of 15.1 in MBL and 14.6 in CCL vs. 11.0 in control) [123]. Higher levels of 8-oxodG in lymphocytes than in urine were correlated with decreases in the capacity of DNA repair systems, while no differences in the oxidative statuses of the MBL and CLL patients suggested that the oxidative injuries occurred during a pre-leukemic state of the disease.

Different studies have reported changes in the 8-oxodG levels in urine induced by various environmental factors: (i) ambient PM2.5 (0.26 nmol/kg in autumn, 0.22 nmol/kg in winter, 0.24 nmol/kg in spring and 0.21 nmol/kg in summer) [84,124], (ii) silica (1.99 ± 0.94 µmol/mol of creatinine in silicotics and 2.28 ± 0.92 µmol/mol of creatinine in exposed workers) [87], (iii) chromium (1149.5 ± 759.5 pmol/kg in exposed workers vs. 730.2 ± 377.6 pmol/kg in controls) [125], (iv) cobalt (1.52 ± 1.69 mol/mol creatinine in individuals exposed to Co and 1.63 ± 1.42 mol/mol creatinine in individuals exposed to hard metals vs. 1.46 ± 1.48 mol/mol creatinine in controls) [126], (v) cobalt and chromium from hip-replacement implants (1.15 µmol/mol of creatinine for implants: 3–4 years; 0.75 µmol/mol of creatinine for implants: 1–2 years) [127], (vi) PAHs (2.6 ± 0.8 g/g of creatinine at the start and 2.5 ± 0.8 g/g of creatinine at the end of the week in roofers exposed to asphalt fume, 2.5 ± 1.7 g/g of creatinine at the start and 3.0 ± 1.7 g/g of creatinine at the end of the week in roofers exposed to coal-tar vs. 3.3 ± 1.9 g/g of creatinine at the start and 3.5 ± 1.7 g/g of creatinine at the end of the week in controls [88]; in engine room personnel of ships, the results were 23.3 nmo/L for individuals with skin contaminated by oil and 18.7 nmol/L for individuals without contaminated skin vs. 18.0 nmol/L for control [128]), (vii) welding fumes (1.7 μg L^−1^/g of creatinine before and 2.1 μg L^−1^/g of creatinine after 3 h of exposure) [129], and (viii) carcinogenic metals including nickel and cadmium [130].

For oxidative stress measurement, saliva has also gained interest as an effective, non-invasive, and easy-to-collect sample source, but it is difficult to obtain accurate 8-oxodG measurements due to its low salivary levels (several pg/mL). The 8-oxoG content in saliva is several hundred-fold higher than that of 8-oxodG, and an average level of 3.80 ng mL^−1^ in normal healthy non-smoking subjects has been detected by HPLC–ECD [131,132].

## 3. Conclusions

The most frequent DNA lesions caused by oxidative stress are 8-oxoG and its nucleotide 8-oxodG (the G and dG oxidation products, respectively), which are regarded as biomarkers for oxidative DNA damage. The analytical quantification of 8-oxoG and 8-oxodG in urine, blood, tissue, and saliva is essential, as it is used to determine body oxidative stress and to evaluate the risk, diagnose in early stages, and predict the consequences of the treatment of various conditions, such as autoimmune, neurodegenerative, cardiovascular, and inflammatory diseases; diabetes; cancer; and other age-related diseases. The present review offers an exhaustive analysis of the most recent developments in HPLC–ECD methodologies for 8-oxoG and 8-oxodG analytical quantification in cellular DNA and in body fluids.

## Data Availability

Not applicable.
